# Double-Stranded RNA Targeting Dicer-Like Genes Compromises the Pathogenicity of *Plasmopara viticola* on Grapevine

**DOI:** 10.3389/fpls.2021.667539

**Published:** 2021-05-18

**Authors:** Zeraye Mehari Haile, Daniel Endale Gebremichael, Luca Capriotti, Barbara Molesini, Francesca Negrini, Marina Collina, Silvia Sabbadini, Bruno Mezzetti, Elena Baraldi

**Affiliations:** ^1^Department of Agricultural and Food Sciences (DISTAL), University of Bologna, Bologna, Italy; ^2^Ethiopian Institute of Agricultural Research (EIAR), Addis Ababa, Ethiopia; ^3^Department of Agricultural, Food and Environmental Sciences, Polytechnic University of Marche, Ancona, Italy; ^4^Department of Biotechnology, University of Verona, Verona, Italy; ^5^Research Group on Food, Nutritional Biochemistry and Health, Universidad Europea del Atlántico, Santander, Spain

**Keywords:** *Dicer-like* genes, double-stranded RNA (dsRNA), *Plasmopara viticola*, spray-induced gene silencing, *Vitis vinifera*

## Abstract

Downy mildew caused by *Plasmopara viticola* is one of the most devastating diseases of grapevine, attacking all green parts of the plant. The damage is severe when the infection at flowering stage is left uncontrolled. *P. viticola* management consumes a significant amount of classical pesticides applied in vineyards, requiring efficient and environmentally safe disease management options. Spray-induced gene silencing (SIGS), through the application of exogenous double-stranded RNA (dsRNA), has shown promising results for the management of diseases in crops. Here, we developed and tested the potential of dsRNA targeting *P. viticola Dicer-like* (*DCL*) genes for SIGS-based crop protection strategy. The exogenous application of *PvDCL1/2* dsRNA, a chimera of *PvDCL1* and *PvDCL2*, highly affected the virulence of *P. viticola*. The reduced expression level of *PvDCL1* and *PvDCL2* transcripts in infected leaves, treated with *PvDCL1/2* dsRNA, was an indication of an active RNA interference mechanism inside the pathogen to compromise its virulence. Besides the protective property, the *PvDCL1/*2 dsRNA also exhibited a curative role by reducing the disease progress rate of already established infection. Our data provide a promising future for *PvDCL1/2* dsRNA as a new generation of RNA-based resistant plants or RNA-based agrochemical for the management of downy mildew disease in grapevine.

## Introduction

Grapevine (*Vitis vinifera* L.) is an important fruit crop cultivated worldwide for fresh and dry fruit consumption and for wine production. Wine production trend is increasing yearly with the world wine trade worth getting about US $40 billion in the year 2018; Italy, France, and Spain being the largest wine-producing countries, contributing half of the world production.^1^ Grapevine production is affected by several pre- and post-harvest pathogens that affect quality during production and processing. Some of the economically important diseases of the crop are gray mold, powdery mildew, and downy mildew caused by *Botrytis cinerea*, *Erysiphe necator*, and *Plasmopara viticola*, respectively. The obligate biotrophic oomycete *P. viticola* attacks all green parts of grapevine, and the damage is severe if the infection occurring during flowering is not managed. Surprisingly, all cultivated European *V. vinifera* cultivars are susceptible to the pathogen (Armijo et al., 2016), which makes the management of downy mildews in vineyard and other crops rely on synthetic fungicides. As a result, its management, together with powdery mildew, consumes about two-thirds of all synthetic fungicides sprayed for disease management of crops in the European Union (Eurostat., 2007). With such heavy reliance on agrochemicals to control *P. viticola*, not only pathogen strains have developed resistance to several fungicides (Gisi and Sierotzki, 2008), but there also exist social concerns about environment and human health, which makes it urgent to find alternative control strategies.

The findings that exogenous small RNAs (sRNA) and double-stranded RNA (dsRNA) trigger posttranscriptional gene silencing (Fire et al., 1998; Hamilton and Baulcombe, 1999) have opened new avenues to exploit the gene silencing mechanism as a new class of regulatory molecules during plant–pathogen interaction. The gene silencing occurs *via* RNA interference (RNAi) machinery, a natural biological process conserved in most eukaryotes where sRNA molecules regulate gene expression by targeting specific endogenous messenger RNA molecules in a sequence-specific manner (Vaucheret and Fagard, 2001; Castel and Martienssen, 2013). The silencing signals of sRNA are bidirectional cross-kingdom, moving from the host to its interacting organism, and *vice versa* (Tomilov et al., 2008; Weiberg et al., 2015; Wang et al., 2016; Cai et al., 2018).

The involvement of sRNAs in the crosstalk between plant hosts and their fungal and oomycete pathogens has also been suggested (Weiberg et al., 2013; Brilli et al., 2018), implying that exploiting the RNAi mechanisms of both the hosts and the pathogens can represent a new strategy in fungal and oomycete disease management. Transgene-derived artificial sRNAs inducing gene silencing, called host-induced gene silencing (HIGS), have been observed providing resistance to plants against fungi (Nowara et al., 2010; Koch et al., 2013; Zhu et al., 2017) and oomycetes (Vega-Arreguin et al., 2014; Jahan et al., 2015). Interestingly, recent findings revealed that the external application of dsRNA also conferred host plant resistance to fungal pathogens by silencing targeted genes (Koch et al., 2016; Wang et al., 2016; McLoughlin et al., 2018; Nerva et al., 2020), an approach referred to as spray-induced gene silencing (SIGS).

The exogenous application of dsRNAs targeting *Dicer-like* (*DCL*), *lanosterol 14*α*-demethylase*, *chitin synthase*, and *elongation factor* genes of *B. cinerea* negatively affected its pathogenicity in multiple hosts (Wang et al., 2016; Nerva et al., 2020). Similarly, spraying of dsRNA targeting three *cytochrome P450* genes of *Fusarium graminearum* inhibited fungal growth at sprayed and distal parts of detached barley leaves (Koch et al., 2016). While these research findings provided proof that SIGS-based plant protection is effective against targeted pathogens, there is also indication that the effects of dsRNA can be reproduced on closely related pathogens based on sequence homology (McLoughlin et al., 2018). According to McLoughlin et al. (2018), dsRNA targeting *SS1G_05899* and *SS1G_02495* genes of *Scelerotinia sclerotiorum*, both involved in redox reaction, restricted the progress of the pathogen on a susceptible *Brassica napus* cultivar. Remarkably, the cultivar was also resistant to *B. cinerea* when treated with dsRNA targeting *BC1G_01592* and *BC1G_04955*, the *B. cinerea* homologs to *SS1G_05899* and *SS1G_02495*, respectively. Such results provide compelling evidence about the adaptability and flexibility of SIGS technology in crop disease management. In this study, we investigate the potential of dsRNA targeting *P. viticola DCL* genes for SIGS-based crop protection strategy. We show that the application of dsRNA targeting *PvDCL1/2* extremely reduces the pathogenicity of *P. viticola* and the expression level of the targeted genes, indicating that RNAi-based control strategy can indeed represent a promising alternative to hazardous agrochemical application to manage downy mildew disease of grapevine.

## Materials and Methods

### Design and Production of dsRNA and Rate of Application

*Plasmopara viticola* genes encoding two Dicer-like proteins, as defined by the presence of a Dicer dimerization domain, corresponding to *PVITv1_T038441* and *PVITv1_T003331*, hereafter referred to as *PvDCL1* and *PvDCL2*, respectively (Brilli et al., 2018), were selected. For RNAi, 258- and 257-bp fragments of *PvDCL1* and *PvDCL2* sequences, respectively (Supplementary Table 1), were chosen as target, and the corresponding chimeric dsRNA molecule (*PvDCL1/2*, 515 bp) was chemically synthesized by AgroRNA (Genolution Inc., Seoul, Republic of Korea; Supplementary Figure 1). DsRNA targeting *B. cinerea DCL 1* and *2* genes, *BcDCL1/2* (490 bp; Wang et al., 2016), produced in the same way, was used as the negative control. After assaying different dsRNA concentrations, 75, 100, or 125 ng μl^–1^ concentrations of dsRNA were used for spot inoculation in a total volume of 50 μl.

### Plant Material and *Plasmopara viticola* Inoculation

Seedlings of *V. vinifera* cv. Trebbiano were raised in growth chamber at 22°C ± 1°C and 12/12 h light cycle. *P. viticola* (strain 465, belonging to University of Bologna collection) was maintained on grapevine leaves at 22°C ± 1°C and 12/12 h of photoperiod. Sporangia were harvested in distilled water and filtered through cheesecloth. Sporangia concentration was determined using hemocytometer.

Fully expanded third and fourth leaves from 6–8-week-old grapevine seedlings were detached and immediately placed on wet absorbing paper in a plastic box. Detached leaves were surface sterilized for 1 min with 70% ethanol and then rinsed three times with sterile water. For assaying dsRNA as preventive treatment, the abaxial side of each leaf was treated with three droplets of 50 μl of either dsRNA or water. After 2 h, 7.5 μl of a 1 × 10^5^ ml^–1^ sporangia solution was placed on top of the droplets. Disease progress was evaluated until 14 days post inoculation (dpi) in five biological replicates. A single leaf was considered a biological replicate.

For assaying dsRNA as curative treatment, each leaf was first challenged by the pathogen by applying four droplets of 7.5 μl of a 1 × 10^5^ ml^–1^ sporangia solution, and after 7 dpi, when a visible sign of *P. viticola* was observed, 50 μl of either dsRNA or water was placed on top of each spot of the progressing pathogen. Disease progress was evaluated until 14 dpi, i.e., 7 days post treatment (dpt) of either dsRNA or water, in three biological replicates.

To assess the progress of the pathogen, leaf area covered by *P. viticola* (in square millimeters) was measured from the digital images using the free software ImageJ program.^2^ Leaf area covered by the pathogen, area under disease progress curve (AUDPC), and disease progress rate data were analyzed using analysis of variance. Means were separated by Tukey’s honestly significant difference test.

### RNA Extraction and Quantitative PCR Analysis

Leaves that were treated in the preventive assay were collected at 7 dpi, in three replicates, immediately frozen in liquid nitrogen, and kept at −80°C until use. RNA was extracted using a rapid cetyltrimethylammonium bromide (CTAB) method (Gambino et al., 2008). First-strand cDNA was synthesized from 1 μg of total RNA, pretreated with TURBO DNA-free Kit^TM^ (Invitrogen, CA, United States), using ImProm-II Reverse Transcriptase (Promega), following the manufacturer’s guide. Quantitative PCR (qPCR) was performed in an MX3000 thermocycler (Stratagene, CA, United States) using 0.25 μl of cDNA and 200 nM of specific forward and reverse primers (Supplementary Table 2) in a total volume of 12.5 μl using Maxima^®^ SYBR Green/ROX qPCR Master Mix (Fermentas). Each amplification reaction was run in duplicate. The cycling parameters were as follows: 5 min at 95°C, 40 cycles of 15 s at 95°C, 25 s at 61°C, and 30 s at 72°C. A melting curve was established from 55°C to 90°C by changing 0.5°C every 10 s. For normalization, *P. viticola* elongation factor eIF1b was used. Each primer pair’s amplification efficiency was calculated using LinReg (Ruijter et al., 2009). The amplification efficiency value obtained was used to calculate the relative quantity (RQ) and normalized RQ (NRQ) according to Hellemans et al. (2007). Statistical analyses of the qPCR results were made after log2(NRQ) transformation (Rieu and Powers, 2009). Statistical significance was calculated by Tukey’s honestly significant difference test.

## Results

### Spray-Induced Gene Silencing of *Plasmopara viticola* DCL Genes Hampers Disease Development

Preliminary inoculation assay was conducted to determine a baseline concentration of *PvDCL1/*2 dsRNA that could affect *P. viticola DCL1* and *DCL2* genes and consequently inhibit its germination and/or colonization of grapevine leaves. After the treatment with 10 and 50 ng μl^–1^ *PvDCL1/2* dsRNA and water, as control, detached grapevine leaves were challenged with *P. viticola* sporangia. Inoculated leaves were monitored for 2 weeks. Sign of *P. viticola* infection was conspicuous around the inoculation spot starting from the fifth dpi, mostly on control and on leaves treated with 10 ng μl^–1^ *PvDCL1/2* dsRNA, where white fluffy growth of sporangiophores and sporangia appeared. At 14 dpi, the rate of disease progress was relatively slower in leaves that received 50 ng μl^–1^ of *PvDCL1/2* dsRNA (Supplementary Figure 2), indicating that pathogen control efficiency can increase with higher concentrations.

**FIGURE 1:**
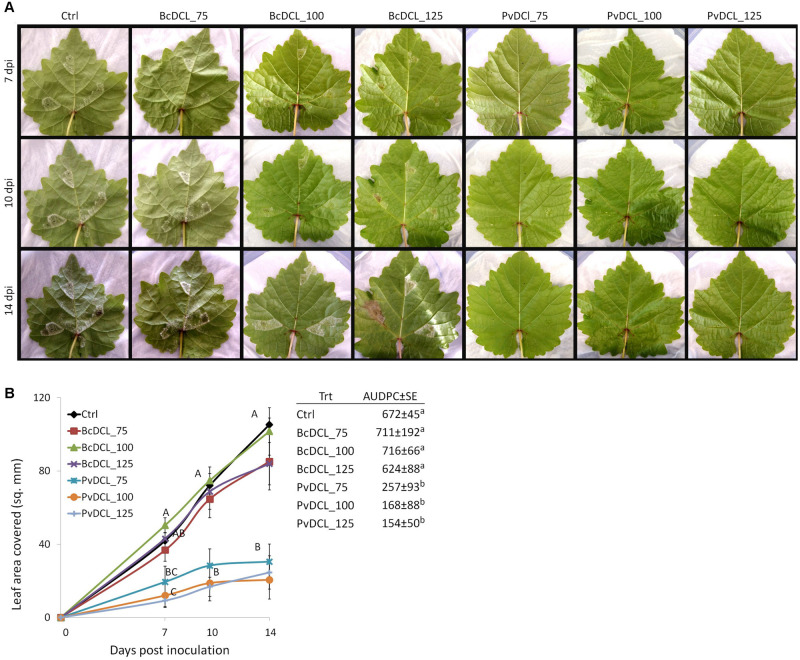
Externally applied *PvDCL1/2* double-stranded RNA (dsRNA) on detached grapevine leaves and *Plasmopara viticola* infection. **(A)** Progress of *P. viticola* on grapevine leaves at 7, 10, and 14 days post inoculation (dpi). Leaves were treated with 50 μl of water (ctrl) or dsRNA [75, 100, or 125 ng μl^– 1^ of dsRNA of *BcDCL1/2* (BcDCL_75/100/125) and *PvDCL1/2* (PvDCL_75/100/125)] before being inoculated with 7.5 μl of a 1 × 10^5^ ml^– 1^ sporangia. **(B)** Disease progression of *P. viticola* expressed as leaf area covered and as area under the disease progress curve (AUDPC ± SE, mm^2^ × day) through 14 dpi. Error bars indicate standard error. Means at each dpi and AUDPC followed by a common letter are significantly not different according to Tukey’s honestly significant difference test (*P* ≤ 0.05).

**FIGURE 2:**
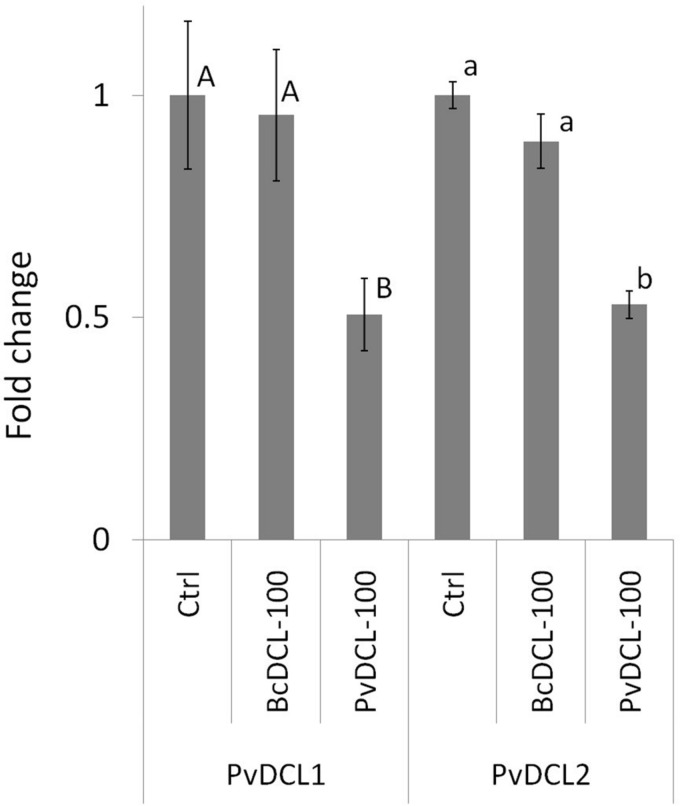
Expression profiles of *PvDCL1* and *PvDCL2* following *Plasmopara viticola* inoculation on leaf samples treated with 50 μl of water (ctrl) or 100 ng μl^– 1^ of double-stranded RNA (dsRNA) of either *BcDCL1/2* (BcDCL-100) or *PvDCL1/2* (PvDCL-100). Gene expression level was determined by quantitative PCR (qPCR). Bars represent fold change of dsRNA-treated sample relative to ctrl sample at 7 days post inoculation. Normalization based on the expression levels of elongation factor, *PveIF1b*, was carried out before calculating fold changes. Error bar represents standard error of the mean of three biological replicates. Expression values followed by a common letter are significantly not different among samples, according to Tukey’s honestly significant difference test (*P* ≤ 0.05), using one-way ANOVA of log2 [normalized relative quantity (NRQ)].

Therefore, the ability of *PvDCL1/*2 dsRNA to control *P. viticola* growth in preventive treatment was assessed using higher concentrations (i.e., 75, 100, and 125 ng μl^–1^). Treatments with *BcDCL1/2* targeting *B. cinerea DCL1* and *DCL2* genes and water were used as controls. As shown in Figure 1A, the fluffy growth of sporangiophores was quite visible on control leaves treated with either water or *BcDCL1/2* dsRNA at the three different concentrations. On the contrary, the pathogen’s progress was substantially low or null on leaves that received *PvDCL1/2* dsRNA. As a consequence, the area covered by *P. viticola* and the AUDPC values at 7, 10, and 14 dpi were significantly and consistently lower in leaves treated with *PvDCL1/2* dsRNA than in those treated with *BcDCL1/2* dsRNA or water (Figure 1B), confirming that *PvDCL1/2* dsRNA hampered *P. viticola* growth. To confirm that the inhibition of *P. viticola* growth by *PvDCL1/2* dsRNA was due to the downregulation of *PvDCL1* and *PvDCL2* genes, their expression, normalized to *P. viticola* elongation factor eIF1b, was quantified at 7 dpi using qPCR. We found that the relative expression of both *PvDCL1* and *PvDCL2* was reduced as compared to the controls (Figure 2). Compared with water and *BcDCL1/2*-treated leaves, the NRQs of *PvDCL1* and *PvDCL2* transcripts in leaves treated with 100 ng μl^–1^ concentration of *PvDCL1/2* dsRNA were reduced on average by 48 and 44%, respectively, which is in line with the concept of RNAi-based sequence-specific silencing *via* SIGS.

### Spray-Induced Gene Silencing of PvDCLs Shows a Curative Effect Against *Plasmopara viticola*

**FIGURE 3:**
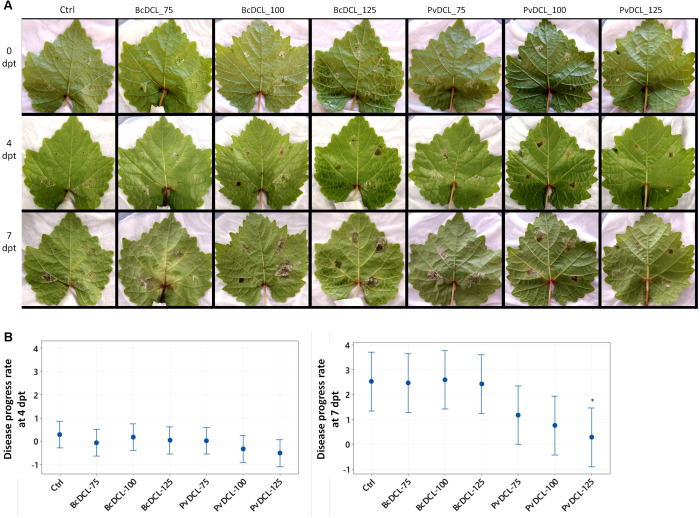
Progress of *Plasmopara viticola on g*rapevine leaves after being treated with *PvDCL1/2* double-stranded RNA (dsRNA). **(A)** Progress of already established *P. viticola* infection after receiving dsRNA treatments. Leaves were treated with 50 μl of water (ctrl) or dsRNA [75, 100, or 125 ng μl^– 1^ of dsRNA of *BcDCL1/2* (BcDCL_75/100/125) or *PvDCL1/2* (PvDCL_75/100/125)] 7 days after being inoculated with 7.5 μl of a 1 × 10^5^ ml^– 1^ sporangia [i.e., 0 days post treatment (dpt) of dsRNA]. **(B)** Disease progress rate at 4 and 7 dpt, computed by taking leaf area covered by *P. viticola* at 7 dpi (0 dpt) as a reference. Bars are 95% confidence interval, and asterisks (^∗^) indicate statistically significant differences according to Tukey’s honestly significant difference test (*P* ≤ 0.05).

The observed protective effect of *PvDCL1/2* dsRNA prompted us to check whether the dsRNA also has a curative effect against *P. viticola*. Detached leaves were initially inoculated with *P. viticola* sporangia, and then, once the infection has been established (i.e., 7 dpi), dsRNA was applied [i.e., the time of either dsRNA or water application is marked as 0 day post treatment (dpt)]. At each inoculation spot, 50 μl of dsRNA or water was added on top of the growing mycelia. At 4 dpt, the progress of the pathogen stagnated in most of the treatments, with more pronounced effect on leaves that received 100 and 125 ng μl^–1^ of *PvDCL1/2* dsRNA (Figure 3A). After 4 dpt, recovering of pathogen growth was more apparent on all leaves. At 7 dpt, the disease advanced more on leaves treated with *BcDCL1/2* and water than on those treated with *PvDCL1/2*, especially at the highest concentration (Figure 3A). Computing the rate of disease progress, taking diseased area at 7 dpi (0 dpt) as a reference, the disease progress rate was relatively slower on leaves treated with *PvDCL1/2*, with more pronounced effect at 7 dpt (Figure 3B). The result shows that the *PvDCL1/2* dsRNA can also hamper the expansion of already established downy mildew disease.

Compared to the preventive application, where all the three concentrations of *PvDCL1/*2 inhibited the growth of the pathogen significantly, when used as curative treatment, the rate of the pathogen growth was reduced significantly only at the highest concentration of *PvDCL1/2* dsRNA. These data show that the exogenously applied dsRNA targeting *PvDCL1/*2 has both promising protective and curative effects.

## Discussion

In grapevine cultivation, downy mildew, caused by *P. viticola*, is among the major diseases requiring repeated applications of pesticides within a growing season. In this study, we show that external application of long non-coding dsRNA, 515 bp long, targeting the two *DCL* genes of *P. viticola* reduced the progress of the pathogen on grapevine leaves. Transcript level reduction of the target genes, *PvDCL1* and *PvDCL2*, suggests specific RNA silencing effect triggered by *PvDCL1/2* dsRNA. To our knowledge, this is the first report showing the potential of exogenously applied RNAi molecules as an effective strategy for oomycete management in crops. The results presented further support the use of SIGS-based strategy for fungal pathogen management (Koch et al., 2016; Wang et al., 2016; McLoughlin et al., 2018; Nerva et al., 2020).

Non-coding sRNA molecules derived from plant pathogens could play a role in suppressing host immunity (Weiberg et al., 2013; Brilli et al., 2018) and hence could be regarded as additional classes of effectors, besides protein coding effector genes studied so far. It has been demonstrated that *B. cinerea* sRNAs (Bc-sRNAs) triggered the silencing of *Arabidopsis* and tomato targets involved in host immunity, such as *mitogen-activated protein kinase 1* (*MPK1*), *MPK2*, *peroxiredoxin*, and *cell wall-associated kinase* genes. Once they have entered the plant cell, Bc-sRNAs hijack the host’s RNAi machinery, binding to Argonaute 1 (AGO1) protein and directing the silencing of host immunity genes (Weiberg et al., 2013). Accordingly, the *ago1* mutant *Arabidopsis* exhibited reduced susceptibility to *B. cinerea*, and the expression of sRNAs that target *B. cinerea DCL1* and *DCL2* in *Arabidopsis* and tomato led to the silencing of the *BcDCL* genes and affected the fungal pathogenicity and growth, also when exogenously applied on different organs and tissues (Weiberg et al., 2013; Wang et al., 2016). In addition, *dcl1 dcl2 B. cinerea* double mutant, which is unable to produce sRNAs, displayed a stunted pathogenicity on several hosts (Weiberg et al., 2013; Wang et al., 2016). In a recent study, it was observed that during *V. vinifera*–*P. viticola* interaction, the sRNA profile of *P. viticola* showed enrichment in 21- and 25-nt sRNAs, which were also abundantly expressed in sporangia (Brilli et al., 2018). According to the study, the presence of DCLs, AGOs, and RNA-dependent RNA polymerase confirms the existence of RNA silencing machinery in *P. viticola*, which is active during its interaction with grapevine (Brilli et al., 2018).

The fact that the external application of *PvDCL1/2* dsRNA extremely reduced the pathogenicity of *P. viticola*, coupled with the observed reduction in *PvDCL1* and *PvDCL2* transcript levels, might suggest that the pathogen can take up external dsRNA and that the RNAi machinery is active during the infection process. Similarly, reduced disease symptoms and sequence-specific silencing of target genes were also observed in *B. cinerea*, *F. graminearum*, and *S. sclerotiorum* (Koch et al., 2016; Wang et al., 2016; McLoughlin et al., 2018), following the external application of dsRNA.

Reduced pathogenicity of plant pathogens due to sRNA and dsRNA has put forward the considerations of RNAi-based technology as a new plant protection method, at least for those pathogens having *bona fide* RNA silencing machinery. *In planta* gene silencing of pathogen target genes, a mechanism known as HIGS, has also been reported (Nowara et al., 2010; Koch et al., 2013; Vega-Arreguin et al., 2014; Jahan et al., 2015; Zhu et al., 2017). Furthermore, for vegetatively propagated crops like grapevine, HIGS can be exploited to obtain RNAi-based rootstocks, which can produce sRNA able to move to a grafted untransformed scion and protect it from pathogen infection, as sRNAs have high mobility between shoot and root (Gouil and Lewsey, 2021; Li et al., 2021). In addition, *in planta* expressed RNAi sequences do not encode for protein products and are designed against specific genes of target pathogens or susceptibility factor without affecting other non-target organisms. All these features together could reduce data requirements for risk assessment of such RNAi-based plants (Limera et al., 2017; Arpaia et al., 2020).

In addition to the HIGS potential application, the results of this research confirm the potential of the gene silencing technology also to develop new RNAi-based fungicides, known as SIGS. To ensure sustainable food production, European Union and global sustainability policies emphasize the need to replace contentious pesticides with safe, efficient, and cost-effective alternatives (Taning et al., 2020). The high selectivity of RNAi-based products, due to sequence-specific modes of action, compared with other conventional pesticides, makes them a promising solution to substitute or reduce reliance on contentious pesticides. Yet there are still relevant aspects to be clarified, such as local and remote translocation and environmental stability of applied sRNAs, before pushing forward SIGS as an alternative solution to toxic pesticides. Despite many solutions reported to stabilize the RNA molecules and make their administration in the field easy and effective, more effort should be taken on the risk assessment studies in order to clarify the risks associated with the use of these molecule for the farmers, consumers, and environment and proceed with the necessary regulatory protocols in order for them to reach the market.

In this study, we demonstrated that dsRNA specifically designed to silence *PvDCL1* and *PvDCL2* genes efficiently controls downy mildew disease caused by *P. viticola* on grapevine, a disease that forces to consume significant amounts of pesticides that are applied every year on vineyards. Although the mechanism behind the uptake and transport of the externally applied dsRNA needs further studies, the presented data give important scientific information on such new-generation RNA-based fungicides, which are environmentally safe and sustainable. So far, externally applied RNAi-based disease suppression data are limited on plant pathogens from Ascomycetes, but with our findings, we extended the possibility of using externally applied dsRNA for managing devastating plant pathogen oomycetes like *Phytophthora* and *Pythium* species.

## Data Availability Statement

The raw data supporting the conclusions of this article will be made available by the authors, without undue reservation.

## Author Contributions

ZH made the experiments and wrote the manuscript. DG made the cloning and plasmid constructs together with LC. BMo and FN contributed to the experiment to confirm gene silencing. MC helped with infections. SS and BMe provided support with the RNAi experiment and participated actively in manuscript writing. EB provided funding and general supervision to the experiments and writing. All authors contributed to the article and approved the submitted version.

## Conflict of Interest

The authors declare that the research was conducted in the absence of any commercial or financial relationships that could be construed as a potential conflict of interest.

## Footnotes

1 http://www.oiv.int/

2 http://imagej.nih.gov/ij/
