# Supplementary material for: Double-Stranded RNA Targeting Dicer-Like Genes Compromises the Pathogenicity of Plasmopara viticola on Grapevine
Source: Front Plant Sci. 2021 May 18;12:667539. doi: 10.3389/fpls.2021.667539 (PMC8167485; doi:10.3389/fpls.2021.667539)
Supplement: Supplementary file 1 [file Data_Sheet_1.PDF]

Supplemental files

Supplemental Table 1. Sequences used for double strand RNA synthesis.

Gene ID	Gene name	Sequence
PVITv1_T038441	*PvDCL1*	ATGATGGACACCTCGTTGTGGGAGCACCAACGGGAGAT<br>CGTGGCTGTGGCGCGACATCGCAGCGTGTTAGTGAGTA<br>GTTCGCAGTCTGTAGGAAAGACGCATGTAAGCTGTGCA<br>CTGCTGTGCGAGGCCGCTGCCTCTAGTCCGAAGCTACAC<br>GCATTGGCGATTGCTGCATCGCCTGTGGGCCGATCGGCT<br>CTACAGACGCAGCTAGCGAGACTGTGTGGACTTCGCGT<br>GCTCTGTAGCGATTCAGACAATGCAAGA
PVITv1_T003331	*PvDCL2*	TAGGCGATACGGGAATCGGCAAAACCTTTCTTGCCATAG<br>CATTATTGTCCGAGCAAGACTACTCGGGCGACCGACGTG<br>CGTTCTTTATGGCTCCGACCCGCCAGTTGGTGGTGCAGA<br>TTACGGCCAAGATTCGCCAGACGAGCACGTTGCGCGTC<br>AATTCGTATTGCGGACGGACAGCTGATTTGTGGGACGCC<br>ACACAGTGGGAACGGGAGCTGCAGCTCACGCGCGTGTT<br>TGTGTGCACACCCGAGATTGTACGC

Supplemental Table 2. List of qPCR primers used. Gene identification, gene name, primer name, and primer sequence are provided.

Gene ID	Gene name	Primer name	Primer sequence
PVITv1_T004162	PveIF1b	PveIF1b_F	ACAACGGTGCAAGGCTTAGC
		PveIF1b_R	ACTCGCGAATGTTAGTCCGC
PVITv1_T038441	PvDCL1	PvDCL1_F	AGCGAGACTGTGTGGACTTC
		PvDCL1_R	GCCTTTTCGCAGCATCTCTT
PVITv1_T003331	PvDCL2	PvDCL2_F	CGGACAGCTGATTTGTGGGA
		PvDCL2_R	GGCACTCGTCAAACACTAGC

.

Supplemental Figure 1. Agarose gel electrophoretic analysis of PvDCL1/2 and BcDCL1/2 dsRNA chemically synthesized by AgroRNA (Genolution Inc., Seoul, Republic of Korea). Samples, diluted 40x were loaded as 5 µL. M: size molecular marker. The quality of the dsRNA, as measured by 260/280 and 260/230 absorbance, was quantified by NanoDrop 1000 Spectrophotometer (Thermo scientific, Waltham, USA).

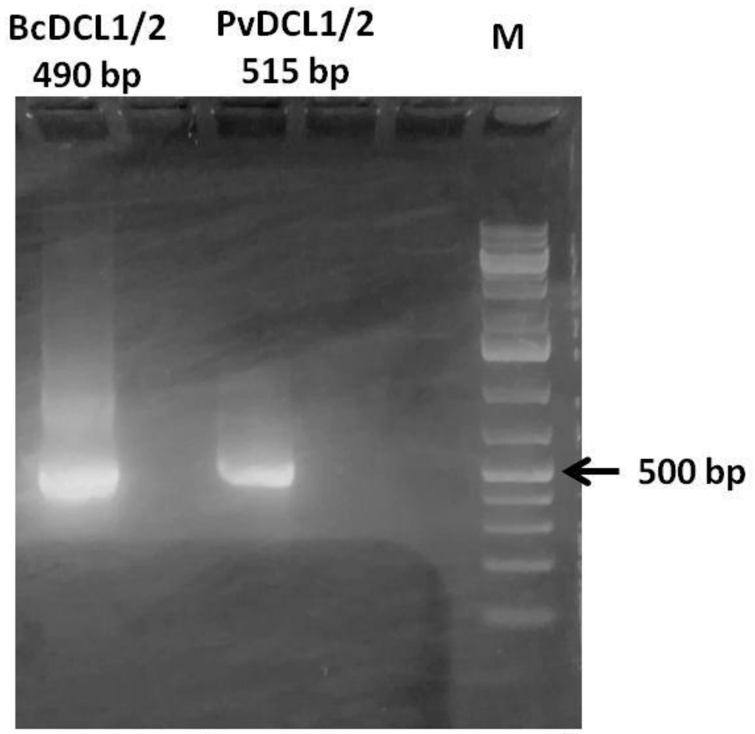

	260/280	260/230
PvDCL1/2	1.84	2.26
BcDCL1/2	2.06	2.41

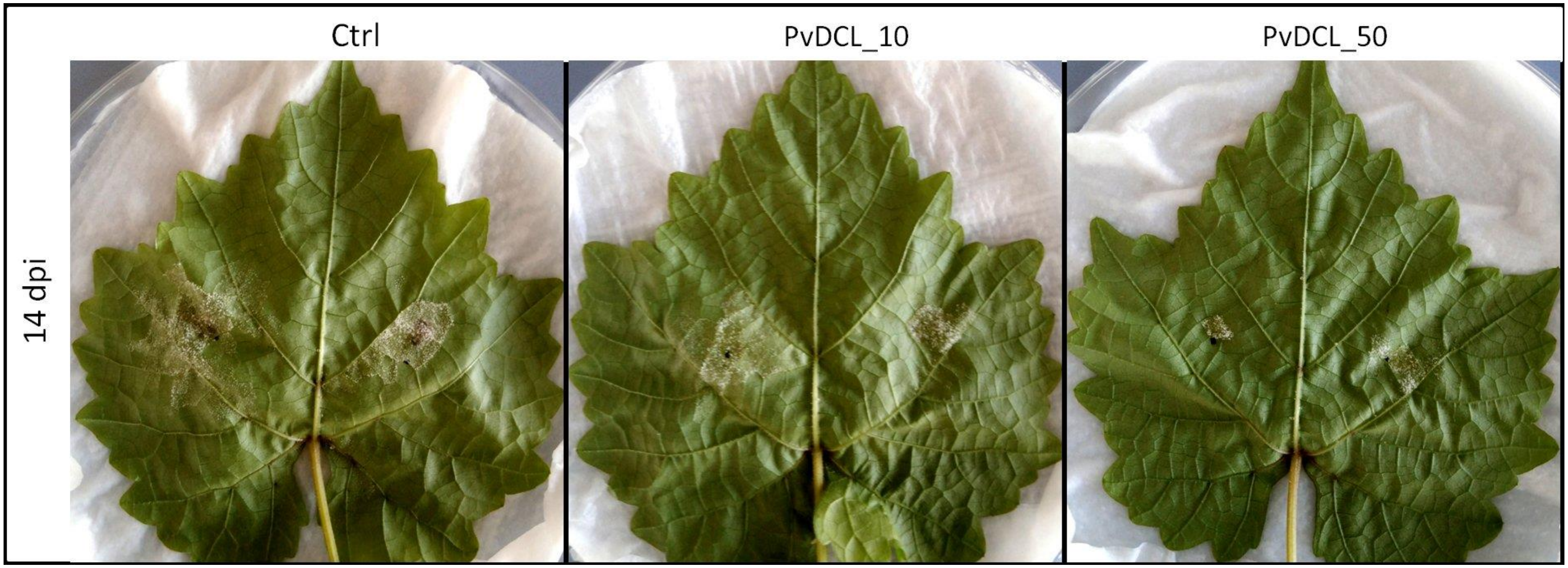

Supplemental Figure 2. Externally applied *PvDCL1/2* dsRNA on detached grapevine leaves inhibited *Plasmopara viticola* infection. Leaves were treated with 50 µl of water (ctrl) or dsRNA before inoculated with 7.5 µL of a 1 $*10^5$ mL^{-1} sporangia. PvDCL-10: PvDCL1/2 dsRNA at 10 ng $µl^{-1}$ concentration; PvDCL-50: PvDCL1/2 dsRNA at 50 ng $µl^{-1}$ concentration; dpi: days post inoculation
